# Isolated Neutropenia as a Sentinel of High-Risk Clonal Evolution: Acute Myeloid Leukemia With Myelodysplasia-Related Changes Harboring *TP53* Deletion via Isochromosome 17q and Deletion 20q Mimicking a Myeloproliferative Neoplasm

**DOI:** 10.14740/jmc5297

**Published:** 2026-04-29

**Authors:** Caryn Louise D. Gutierrez, Anne Kristine H. Quero-Taggaoa

**Affiliations:** a Division of Hematology, Department of Internal Medicine, Philippine General Hospital, Manila, Philippines

**Keywords:** Isolated neutropenia, Clonal evolution, Acute myeloid leukemia with myelodysplasia related changes, *TP53* deletion, Isochromosome 17q, Deletion 20q, Leukoerythroblastosis, High-risk cytogenetics

## Abstract

Isolated neutropenia is often deemed benign in elderly patients, frequently attributed to age-related marrow changes, medications, or nutritional deficiencies. However, persistent and unexplained neutropenia may signal early clonal hematopoiesis or evolving myeloid malignancy. While acute myeloid leukemia with myelodysplasia-related changes (AML-MRC) usually arises from multilineage cytopenia, presentation with isolated neutropenia and myeloproliferative neoplasia (MPN)-like features is exceedingly rare. This case underscores the importance of early, risk-adapted assessment in such scenarios. A 69-year-old woman presented with isolated mild neutropenia (absolute neutrophil count (ANC) 1,188/µL) and easy bruising. With no other cytopenias or symptoms, bone marrow biopsy was deferred. Six months later, she developed abdominal discomfort, anorexia, and massive splenomegaly. Laboratory tests revealed leukocytosis with left shift, anemia, thrombocytopenia, and nucleated red cells. Bone marrow biopsy showed > 40% myeloblasts, trilineage dysplasia, and mild fibrosis. *JAK2* mutation and *BCR-ABL* were negative. Cytogenetics demonstrated i(17q) and del(20q); Fluorescence *in situ* hybridization (FISH) showed *TP53* deletion in 81.91% of nuclei, establishing AML-MRC with high-risk features. She was started on azacitidine and venetoclax. Day 28 marrow showed minimal residual disease (blast 8%) but no metaphase growth prompting treatment continuation. She is currently on her fourth cycle with treatment-related transfusion-dependent cytopenias, prompting dose reduction. This case challenges the prevailing “watch-and-wait” paradigm in the evaluation of isolated neutropenia, particularly in older adults. It exposes the hidden risk of clinical inertia, where the absence of other overt cytopenias or symptoms may lead to missed opportunities for early diagnosis of high-risk clonal evolution. In resource-limited setting, this case underscores urgent need for heightened clinical vigilance. Likewise, it highlights the urgent need for a more risk-adapted approach to persistent isolated neutropenia and compels us to confront a critical question: how many patients are we failing to diagnose in time, and how many windows for potentially life-altering intervention are we silently allowing to close?

## Introduction

Isolated neutropenia is frequently overlooked in clinical practice, particularly among elderly patients, where it is often attributed to benign causes such as medications, infections, nutritional deficiencies (e.g., folate and vitamin B12), immune-mediated conditions, or age-related causes [1, 2]. In the absence of additional cytopenias or overt morphologic dysplasia, this subtle manifestation often leads to a delayed or deferred diagnostic workup [3, 4]. In such contexts, especially in a resource-constrained setting, stable patients with persistent isolated neutropenia are frequently managed conservatively, with a “watch and wait” approach grounded in the assumption that the risk of malignant evolution is low [5]. Persistent isolated neutropenia without an identifiable cause may represent the earliest clinical footprint of a clonal myeloid disorder. When persistent, severe and unexplained, isolated neutropenia, although uncommon, may serve as an early harbinger of clonal hematopoiesis or a pre-malignant myeloid condition such as myelodysplastic syndrome (MDS) or clonal cytopenia of undetermined significance (CCUS) [2, 3, 6]. Isolated neutropenia in MDS is rare occurring in only about 2% of cases [7]. Even when these clonal precursors are identified, the current paradigm continues to favor observation over intervention [6, 7].

Acute myeloid leukemia with myelodysplasia-related changes (AML-MRC) is one of the more commonly encountered subtypes of AML, accounting for approximately 20% of all cases [8]. These patients generally have poorer prognosis and demonstrate lower response rates to therapy particularly when associated with high-risk cytogenetic abnormalities such as *TP53* deletion [8, 9]. AML-MRC is defined by > 20% bone marrow blasts plus either a history of MDS/myeloproliferative neoplasia (MPN), MDS-related cytogenetics, or ≥ 50% multilineage dysplasia. Prior chemotherapy, radiation, or recurrent AML-defining cytogenetics exclude the diagnosis [8, 10]. In general, AML-MRC evolves from an antecedent hematologic condition, or overt cytopenias [10, 11]. Considering these points, it raises a crucial yet unresolved question in our current hematologic practice: should we intervene early in cases of unexplained neutropenia, or continue to observe under the prevailing “watch and wait” strategy?

We present a rare case that challenges this paradigm: a case of AML-MRC in an elderly female who initially presented with subtle isolated neutropenia and was closely observed, but subsequently developed aggressive AML with massive splenomegaly, leukoerythroblastosis, and mild marrow fibrosis—features more typical of primary myelofibrosis (PMF) or MDS/MPN overlap syndrome. Cytogenetic analysis revealed an extremely rare coexistence of i(17q) and del(20q), supporting the presence of high-risk clonal evolution. To our knowledge, this is the first documented case of isolated neutropenia progressing to AML-MRC with MPN-like features. This underscores the importance of clinical vigilance and re-evaluation of current thresholds for invasive diagnostics in otherwise stable patients. It also raises important questions about whether early therapeutic intervention might have altered the trajectory of disease progression in cases of isolated neutropenia—a consideration particularly relevant in settings where delayed diagnosis is often the rule rather than the exception.

## Case Report

A 69-year-old female initially presented with occasional episodes of easy bruising in the absence of trauma. Her initial complete blood count (CBC) was notable only for mild neutropenia, with a hemoglobin of 125 g/L, hematocrit 42%, white blood cell (WBC) count of 3.6 × 10^9^/L, absolute neutrophil count (ANC) of 1,188/µL, and platelet count of 217 × 10^9^/L. Coagulation studies were within normal limits. She was diagnosed with senile purpura, with an International Society on Thrombosis and Hemostasis–Bleeding Assessment Tool (ISTH-BAT) score of 2. She was advised to undergo routine monitoring in the outpatient department every 3 months.

At her second follow-up, the patient denied recurrence of easy bruising; however, repeat CBC showed persistent but slight worsening of neutropenia: WBC 2.5 × 10^9^/L, ANC 803/µL, hemoglobin 129 g/L, hematocrit 43%, and a platelet count of 437 × 10^9^/L. Peripheral blood smear (PBS) was performed, revealing a normocytic, normochromic red blood cells (RBCs), and there was a note of presence of occasional polymorphonuclear cell (PMNs) with hyposegmentation and hypogranulation. MDS was considered, and she was advised to undergo bone marrow aspiration (BMA) and biopsy. However, given the absence of transfusion requirements, additional cytopenias, or indications for an immediate change in therapeutic management, the patient declined the biopsy at that time and chose to continue with periodic laboratory monitoring instead. She was advised to do CBC monitoring every 2 months.

At her third follow-up visit, CBC showed slightly improved leukocyte counts but persistent mild neutropenia (WBC 3.2 × 10^9^/L, ANC 1,088/µL), with no anemia (hemoglobin 126 g/L) or thrombocytopenia (platelet count 163 × 10^9^/L). Although still within the normal reference range, the platelet count represented a decline from the previously elevated value of 437 × 10^9^/L. PBS findings remained consistent with prior examination, demonstrating occasional hyposegmented and hypogranulated PMNs without other significant abnormalities. The patient reported new-onset bloating and anorexia. However, abdominal examination remained unremarkable, with no palpable hepatosplenomegaly. BMA with biopsy was offered, but she did not provide consent for the procedure. Peripheral blood MDS fluorescence *in situ* hybridization (FISH) testing was subsequently proposed. At that time, the patient was unable to undergo the test due to financial constraints but indicated willingness to proceed once resources became available.

**Figure 1:**
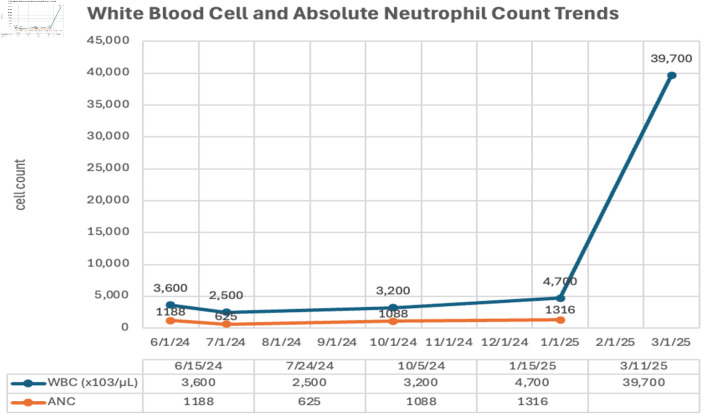
White blood cell (WBC) count and absolute neutrophil count (ANC) trends on outpatient follow-up visits.

**Figure 2:**
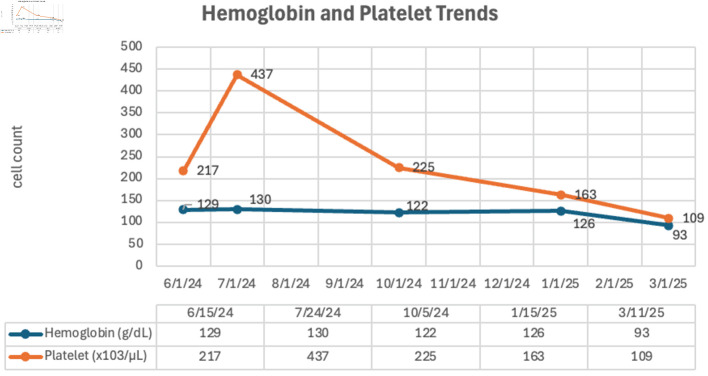
Hemoglobin and platelet trends on outpatient follow-up visits.

One month later, the patient presented to the emergency department with generalized weakness and abdominal pain. On abdominal examination, the spleen was palpable 14 cm below the left subcostal margin and extending beyond midline. Repeat CBC revealed marked leukocytosis (WBC 39.7 × 10^9^/L) with a left-shifted differential: neutrophils 41%, lymphocytes 8%, monocytes 2%, band forms 3%, metamyelocytes 1%, myelocytes 7%, promyelocytes 10%, and blasts 28%. Anemia (hemoglobin 92 g/L) and thrombocytopenia (platelets 109 × 10^9^/L) were also noted. PBS at this time revealed normocytic, normochromic RBCs along with the presence of nucleated red blood cells (nRBCs), and some dacryocytes. There were also occasional dysplastic PMNs characterized by hyposegmentation and hypogranulation. Whole abdominal ultrasound confirmed massive splenomegaly. Figure 1 shows the WBC count, and ANC trends of the patient during subsequent follow-ups, while Figure 2 shows the hemoglobin and platelet count trends.

### Investigations

Given the combination of massive splenomegaly, and leukoerythroblastosis, MDS/MPN overlap with leukemic conversion was initially considered. However, further molecular workup, including *BCR-ABL1* and *JAK2* mutation testing, returned negative.

BMA and biopsy revealed a markedly hypercellular marrow for age (90% cellularity), with sheets of mononuclear cells exhibiting round to oval nuclei, fine chromatin, occasional prominent nucleoli, and high nuclear-to-cytoplasmic ratios, comprising 41–50% of marrow cellularity—consistent with myeloblasts. Trilineage dysplasia was observed, along with mild reticulin fibrosis. These findings were diagnostic of AML. Flow cytometry using a basic leukemia panel confirmed the diagnosis.

**Figure 3:**
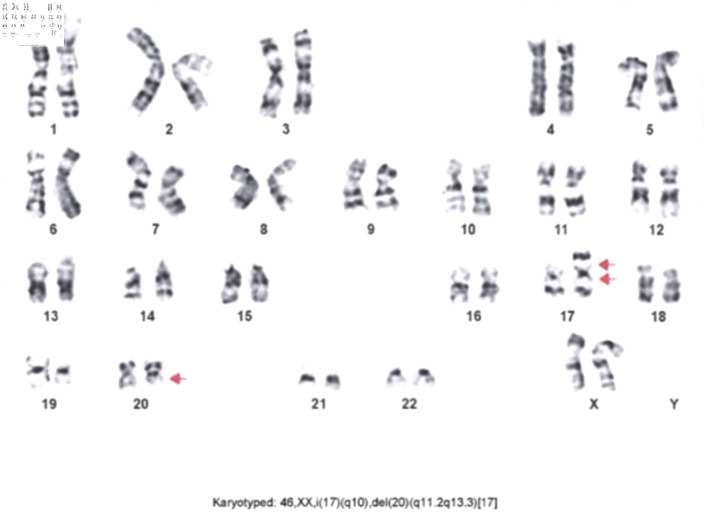
Cytogenetic study showing isochromosome 17q and deletion 20q.

Cytogenetic analysis revealed isochromosome 17q and deletion of the long arm of chromosome 20 (del(20q)) in all 17 metaphase cells examined (Fig. 3). FISH showed a *TP53* deletion in 81.91% of nuclei. *FLT3* mutation was negative, and next-generation sequencing (NGS) was not performed due to financial constraints.

### Treatment

She was ultimately diagnosed with AML-MRC, harboring high-risk cytogenetic features. She was initiated on combination therapy with azacitidine and venetoclax. Day 28 bone marrow assessment following the first cycle showed minimal residual disease, and the repeat karyotyping showed no metaphase growth. This prompted continuation of treatment. However, she subsequently developed transfusion-requiring severe thrombocytopenia and severe anemia, necessitating dose modifications: azacitidine was reduced to 50% of the original dose, and venetoclax was adjusted to 100 mg every other day.

### Follow-up and outcomes

At the time of writing, approximately 1 year after diagnosis, the patient was transitioned from azacitidine plus venetoclax to decitabine (10-day schedule) combined with venetoclax for 14 days due to persistent transfusion dependence. She is currently receiving her second cycle of this regimen with close monitoring for hematologic toxicity and treatment response. Early follow-up has demonstrated a notable reduction in transfusion requirements, suggesting clinical benefit from the modified therapy.

## Discussion

This case highlights the clinical significance of persistent isolated neutropenia as a potential early manifestation of high-risk clonal evolution, culminating in a rare and diagnostically challenging presentation of AML-MRC with MDS/MPN-like features. It reinforces the need for a paradigm shift in how mild, unexplained neutropenia—particularly in older adults—is approached in clinical practice.

In one study by Njue et al, among patients with chronic neutropenia, the majority had idiopathic (50%) or autoimmune (33%) etiologies, while a smaller proportion had congenital forms (17%) [12]. MDS and AML rarely present as isolated neutropenia and are present only in about 2% of the cases [7, 13]. As a result, patients with isolated neutropenia are commonly monitored without extensive investigation, particularly in resource-limited settings where access to diagnostic tools is constrained. However, this conservative approach may delay recognition of clonal hematopoietic disorders, especially in cases where neutropenia is persistent and unexplained. In the present case, neutropenia remained the sole hematologic abnormality for several months, which delayed further investigation and ultimately allowed a high-risk myeloid neoplasm to progress undetected.

Emerging data underscore the importance of reassessing this approach, especially in the context of clonal hematopoiesis of indeterminate potential (CHIP) and CCUS [13]. These clonal states are more common in the elderly [14, 15]. Even in a well-resourced setting, earlier bone marrow examination and molecular profiling might have revealed the presence of *TP53* mutation or other high-risk features; however, the recommendation for these diseases will still focus on closer surveillance without initiating treatment [16]. These modalities are often unavailable or cost-prohibitive in resource-constrained settings. This case powerfully demonstrates how isolated neutropenia can act as a sentinel event in clonal evolution, preceding overt leukemic transformation. The patient’s subsequent presentation with massive splenomegaly, leukoerythroblastosis, and high-risk cytogenetics (*TP53* deletion via isochromosome 17q and del(20q)) exemplifies how an aggressive disease trajectory can go unrecognized in the absence of early suspicion.

In 2008, the World Health Organization (WHO) formally recognized AML-MRC as a distinct subtype within its classification system [10]. AML-MRC is generally considered biologically linked to MDS and was previously referred to as refractory anemia with excess blasts in transformation (RAEB-T) under the French-American-British (FAB) classification [17]. As shown in Table 1, the diagnosis of AML-MRC requires a bone marrow blast percentage exceeding 20% along with at least one of the following: a documented history of MDS or MPN, MDS-associated cytogenetic abnormalities, or dysplasia involving 50% or more of cells in at least two hematopoietic lineages [10, 17]. In this patient, AML-MRC was diagnosed based on the presence of > 40% blasts, multilineage dysplasia, and MDS-related cytogenetic abnormalities, specifically isochromosome 17q (i(17q)), and deletion 20q.

**Table 1:** Diagnostic Criteria for AML-MRC

Diagnosis of AML -MRC according to the following 3 criteria:
≥ 20% bone marrow blasts
At least one of the following
Antecedent MDS-MPN
MDS-related cytogenetic aberration^a^
Multilineage dysplasia ≥ 50%
Exclusion of the following:
Antecedent radiation of cytotoxic therapy
Recurrent cytogenetic aberration defining AML with recurrence cytogenetic aberrations

^a^Myelodysplasia-related cytogenetic aberrations include: 1) complex karyotype (≥ 3 abnormalities); 2) unbalanced abnormalities: loss of chromosome 7 or del (7q), del(5q) or t(5q), isochromosome 17q or t(17p), loss of chromosome 13 or del(13q), del(11q), del(12p) or t(12p), idic(X)(q13); and 3) balanced abnormalities: t(11;16)(q23.3;p13.3), t(3;21)(q26.2;q22.1), t(1;3)(p36.3;q21.2), t(2;11)(p21;q23.3), t(5;12)(q32;p13.2), t(5;7)(q32;q11.2), t(5;17)(q32;p13.2), t(5;10)(q32;q21), t(3;5)(q25.3;q35.1). AML-MRC: acute myeloid leukemia with myelodysplasia-related changes; MDS: myelodysplastic syndrome; MPN: myeloproliferative neoplasia.

Prognostically, AML-MRC represents one of the highest-risk AML subtypes. Multiple studies, including large-scale analyses by Takeda et al and the European LeukemiaNet (ELN), consistently show inferior complete remission (CR) rates, higher relapse risk, and shorter overall survival, particularly in patients with adverse cytogenetics such as *TP53* mutation/deletion or complex karyotypes [18, 19].

The clinical presentation of this case was complicated by features more typically associated with MPNs, namely massive splenomegaly, mild marrow fibrosis, and a leukoerythroblastic blood smear. These findings are unusual in AML-MRC, which more often presents with cytopenias, dysplasia, and a hypercellular marrow without significant fibrosis or extramedullary hematopoiesis. Massive splenomegaly and leukoerythroblastosis are classical hallmarks of PMF and other chronic MPNs, reflecting extramedullary hematopoiesis and marrow exhaustion [20, 21]. The presence of reticulin fibrosis in the marrow further supported an MPN-like phenotype. However, in the absence of common MPN-associated mutations such as *JAK2* V617F, and with negative *BCR-ABL1*, classical MPNs such as PMF, polycythemia vera (PV), essential thrombocythemia (ET), and chronic myeloid leukemia (CML) were ruled out, and the absence of monocytosis or basophilia helped exclude chronic myelomonocytic leukemia (CMML) and atypical CML (aCML) [22, 23].

The cytogenetic profile in this patient was particularly striking and highlights the aggressive biology underlying her disease. Isochromosome 17q is a rare structural chromosomal abnormality formed by duplication of the long arm and simultaneous loss of the short arm of chromosome 17, resulting in trisomy of 17q and monosomy of 17p. Importantly, the short arm of chromosome 17 (17p) harbors the *TP53* tumor suppressor gene. Thus, i(17q) results in loss of *TP53*, either through monosomy 17p or through compound lesions with *TP53* mutations or deletions. The leukemogenic potential of i(17q) is thought to stem from both the loss of tumor suppressor function (via *TP53* haploinsufficiency or deletion) and the gain of dosage-sensitive genes on 17q, such as *MSI2* or the granulocyte colony-stimulating factor receptor (G-CSFR), which promote myeloid proliferation [24]. In AML, i(17q) is found in approximately 0.5–1% of cases and is strongly associated with poor prognosis, refractory disease, and short overall survival, especially when combined with other cytogenetic abnormalities or *TP53* disruption [25, 26]. In our patient, the presence of i(17q) in all 17 metaphases examined suggests a dominant clone, supporting its role as a primary driver of disease biology. The identification of *TP53* deletion in > 80% of interphase nuclei by FISH in this patient represents a critical prognostic determinant. *TP53* alterations are associated with poor response to chemotherapy (especially to anthracyclines and cytarabine), low rates of CR, high relapse rates, and median overall survival often less than 6 months, even with aggressive therapy or allogeneic stem cell transplantation [27, 28].

On the other hand, deletion of the long arm of chromosome 20 is a well-characterized cytogenetic abnormality in MDS, MPN, and less frequently in AML. It is often considered a favorable-risk lesion when isolated, but its prognostic impact changes dramatically when found in association with other abnormalities, particularly complex karyotypes [29]. The minimal commonly deleted region (MCDR) in 20q includes the *L3MBTL1* gene, which acts as a chromatin modifier and tumor suppressor. Its loss has been implicated in ineffective hematopoiesis and progression to AML [30]. In this case, the coexistence of del(20q) with i(17q) suggests a multi-hit pathogenesis, where del(20q) may represent an early lesion in a pre-leukemic clone, followed by acquisition of i(17q) and *TP53* deletion that drives transformation to overt AML. Interestingly, to our knowledge, there are no reported cases in the literature describing the co-occurrence of i(17q) and del(20q). The rarity of this cytogenetic profile makes this case both reportable and educational for the hematology community.

The use of hypomethylating agents (HMAs) plus venetoclax in this patient aligns with emerging data suggesting some benefit in *TP53*-mutated AML, though responses are often transient [31, 32]. Studies such as the VIALE-A trial demonstrated a median overall survival of just 5–7 months in *TP53*-mutated patients receiving azacitidine-venetoclax, highlighting the urgent need for novel strategies in this subset [33].

Beyond its cytogenetic and phenotypic complexity, this case also draws attention to the challenges of timely diagnosis—especially in resource-limited settings, where access to bone marrow biopsy, cytogenetic studies, and NGS is often constrained. In such contexts, patients presenting with persistent but stable cytopenias may not undergo adequate evaluation until transformation to overt leukemia occurs, missing a crucial window for early diagnosis or intervention. The prevailing “watchful waiting” paradigm for isolated neutropenia may thus delay recognition of CHIP or CCUS—entities now acknowledged as precursors to myeloid malignancies and frequently associated with high-risk mutations such as *TP53*, *ASXL1*, *RUNX1*, and *SRSF2* [14, 34]. Importantly, however, even in well-resourced settings, the current standard of care for patients with CCUS remains close monitoring rather than early therapeutic intervention, due to the limited evidence that early treatment alters the natural history of disease or improves outcomes [34, 35]. Thus, despite growing molecular insight into clonal evolution, both systemic barriers in low-resource environments and the absence of established interventional guidelines in high-resource centers contribute to diagnostic inertia—underscoring the urgent need for risk-adapted strategies that can identify patients who might benefit from earlier evaluation and possibly pre-emptive therapy.

This case reinforces the need to reconsider current thresholds for invasive diagnostics in older patients with persistent neutropenia, even when other blood indices are preserved. It demonstrates that isolated neutropenia can represent not just an innocuous laboratory finding but a sentinel event in the trajectory of clonal evolution toward aggressive AML. The presence of MPN-like clinical features further illustrates the overlapping spectrum of myeloid neoplasms and underscores the critical role of integrating morphologic, cytogenetic, and molecular diagnostics in navigating these blurred boundaries.

### Learning points

Persistent isolated neutropenia in older adults warrants careful evaluation, as it may represent early clonal hematopoiesis or evolving myeloid neoplasm rather than a benign finding.

Clinical inertia should be avoided when neutropenia persists without clear etiology, even in the absence of other cytopenias or symptoms.

Myeloid neoplasms may initially present with subtle hematologic abnormalities, and progression to AML with myelodysplasia-related changes can occur after a prolonged indolent phase.

Limited access to bone marrow, cytogenetic, and molecular testing in resource-constrained settings may delay diagnosis and worsen outcomes in patients with evolving clonal hematologic disease.

A risk-adapted and vigilant approach to unexplained neutropenia may facilitate earlier diagnosis and timely intervention.

Careful review of the PBS by an experienced morphologist is essential for detecting early dysplastic changes.

## Data Availability

All data supporting the findings of this case report are included within the article.

## Abbreviations

aCML: atypical chronic myeloid leukemia
AML: acute myeloid leukemia
AML-MRC: acute myeloid leukemia with myelodysplasia-related changes
ANC: absolute neutrophil count
ISTH-BAT: International Society on Thrombosis and Hemostasis–Bleeding Assessment Tool
*BCR-ABL1*: *BCR-ABL1* fusion gene (Philadelphia chromosome)
CBC: complete blood count
CCUS: clonal cytopenia of undetermined significance
CHIP: clonal hematopoiesis of indeterminate potential
CML: chronic myeloid leukemia
CMML: chronic myelomonocytic leukemia
CR: complete remission
del(20q): deletion of the long arm of chromosome 20
ELN: European LeukemiaNet
ET: essential thrombocythemia
FAB: French-American-British classification
FISH: fluorescence *in situ* hybridization
FLT3: FMS-like tyrosine kinase 3
HMA: hypomethylating agent
i(17q): isochromosome 17q
JAK2: Janus kinase 2
MCDR: minimal commonly deleted region
MDS: myelodysplastic syndrome
MDS/MPN: myelodysplastic/myeloproliferative neoplasm
MPN: myeloproliferative neoplasm
NGS: next-generation sequencing
nRBCs: nucleated red blood cells
PMF: primary myelofibrosis
PV: polycythemia vera
RAEB-T: refractory anemia with excess blasts in transformation
TP53: tumor protein p53 gene
WBC: white blood cell
WHO: World Health Organization
